# Changes of Land Use and Land Cover with the Diversity of Fishes, Aquatic Plants, and Bird's Species at Wetland Ecosystem

**DOI:** 10.1155/2021/7533119

**Published:** 2021-12-08

**Authors:** Mohammad Zahangeer Alam, Md. Abdullahil Baki Bhuiyan, Hasan Muhammad Abdullah, Suma Rani Ghosh, Mohammad Maksudul Hassan, Ruzina Akter, M. Rokonuzzaman, Mohammad Shah Alam

**Affiliations:** ^1^Department of Environmental Science, Faculty of Agriculture, Bangabandhu Sheikh Mujibur Rahman Agricultural University (BSMRAU), Gazipur-1706, Bangladesh; ^2^Department of Plant Pathology, Faculty of Agriculture, Bangabandhu Sheikh Mujibur Rahman Agricultural University (BSMRAU), Gazipur-1706, Bangladesh; ^3^Department of Agroforestry and Environment, Faculty of Agriculture, Bangabandhu Sheikh Mujibur Rahman Agricultural University (BSMRAU), Gazipur-1706, Bangladesh; ^4^Department of Environmental Science, Faculty of Agriculture, Bangladesh Agricultural University, Mymensingh-2202, Bangladesh; ^5^Department of Criminology and Police Science, Mawlana Bhashani Science and Technology University, Santosh, Tangail-1902, Bangladesh; ^6^Department of Agricultural Extension and Rural Development, Faculty of Agriculture, Bangabandhu Sheikh Mujibur Rahman Agricultural University (BSMRAU), Gazipur-1706, Bangladesh; ^7^Department of Anatomy and Histology, Bangabandhu Sheikh Mujibur Rahman Agricultural University, Gazipur-1706, Bangladesh

## Abstract

Bangladesh is rich in wetland biodiversity with aquatic plants, fishes, and birds. Mohanganj Upazila is known as the capital of lower Bangladesh. The present study focuses on the changes of land use and land cover (LULC) with a diversity of species that are being least concerned (LC), vulnerable (VU), and endangered (EN). Over the last two decades, the wetland species of Mohanganj were gradually declined. Our results showed that 19 fish, 4 aquatic plants, and 7 bird species were LC in 2015. Among the fish and aquatic plant species, 6 fish species (*Wallago attu, Ompok pabda, Channa punctate, Chitala chitala, Salmostoma phulo,* and *Corica soborna*) and 2 aquatic plant species (*Nymphaea nouchali* and *Nymphaea lotus*) were VU during the dry and rainy season of 2017 and 2019, respectively. In the dry season of 2019, 4 fish species (*W. attu*, *O. pabda, C. punctate*, and *Ch. chitala),* 2 aquatic plant species (*N. nouchali* and *N. lotus*), and 7 bird species (*Anas platyrhynchos, Ardeola grayii, Gyps bengalensis, Alcedo atthis, Phalacrocorax fuscicollis, Porphyrio porphyria,* and *Larus ridibundus*) were EN. Among the species, *W. attu*, *N. nouchaii, G. bengalensis, P. porphyria,* and *L. ridibundus* were extremely endangered categories. Changes in LULC, the establishment of settlements for the increasing population, indiscriminate use of pesticides, environmental pollutions, and climate change are the potential reasons for declining trends of wetland biodiversity. Stern actions on land use policy, expansion of organic agriculture, bioremediation of industrial effluents, and adoption of sustainable environmental policies should be taken by the Government of Bangladesh for immediate conservation of wetland biodiversity.

## 1. Introduction

Wetlands are one of the key natural resources where diversified species live. These wetlands provide natural ecosystem services, such as water, fish, edible food, wood, energy, and recreational activities to human beings. Marsh, fen, and peatland are the most productive wetland ecosystem in the world [1]. Wetlands connect land with water, which may be permanent or temporary, static or flowing, and fresh or brackish, including the areas of marine water. Wetland resources are key to sustainable livelihoods through the process of nutrient and carbon cycles, hydrological cycles, soil-forming dynamics, natural-resource-driven livelihoods, and reducing the vulnerability of crops to pests, disease, drought, and flooding [2, 3]. Sundarbans is the most recognized saltwater wetland with a mangrove forest in Bangladesh [4]. However, Tanguar Haor and Hakaluki Haor are the most remarkable freshwater wetland ecosystems [4, 5].

Sundarbans is exclusively important for the home of the critically endangered Royal Bengal Tiger and Freshwater dolphins [6]. About 137 freshwater fish species and 558 animal species are available in Tanguar and Hakaluki haor, respectively. Out of 1218 vertebrate species in Bangladesh, about 691 species live in wetlands [6]. Bangladesh is rich in wetland biodiversity with 280 freshwaters, 49 amphibians, 160 reptiles, 208 aquatic birds, and 490 marine species present in different wetland ecosystems [7–10]. Most of the species in wetland ecosystems are categorized as rare, endangered, threatened, and vulnerable [11].

The biodiversity of the wetland ecosystem varies from region to continent. It encompasses a range of living things with different habitats. Wetlands are the most valuable ecosystems in the world. It is the rich source of global biodiversity within the major climatic belts due to the evolved collection of fishes, animals, and plants [12]. The wetland ecosystems are surrounded by water either fresh or salty [13]. However, land-based terrestrial ecosystems are found on lands such as forests or grasslands [14]. Therefore, the challenges of these ecosystems are very different.

Globally, there are many major challenges of wetland habitats, such as inadequate social and political capacity, climate change, and insufficient planning by the government to the conservation of species [15]. In Bangladesh, the vulnerability of wetland species and ecosystem services has increased due to the agricultural land conversion, changes in land use and land cover (LULC), deforestation, climate change, harvesting of natural resources, and the introduction of alien species [16]. The depletion of the wetland biodiversity also depends on the wetland type and ecosystem services. Most of these challenges can be overcome through the development of a plant or animal's distinctive behavior [9].

Although some species in wetland ecosystems are capable of overcoming environmental threats, the majority of the species are vulnerable to the changing environment in the world [1, 17]. Sedimentation and flooding are the major causes of reducing species diversity in the wetland ecosystem [18, 19]. Recently, some species disappeared, such as Catarina Pupfish, Sumatran Rhino, Chinese paddlefish, Yangtze giant softshell turtle, Indian Cheetah, Spix Macaw, and Indochinese tiger from the wetland habitats throughout the world [10, 20].

Globally, a quarter of mammals and aquatic species in wetland ecosystems are threatened by human activities over the last 100 years [21, 22]. In Bangladesh, wetland species are exposed to rapid degradation due to high population density, unplanned industrialization and urbanization, habitat destruction, wastewater disposal, and natural hazards [23]. Thus, the biodiversity of wetland ecosystems in Bangladesh has been lost over the last two decades, which creates negative impacts on natural resources, human livelihood, and sustainability [21].

Mohanganj is one of the wetland-based Upazila under the Netrokona district. Netrokona is situated in the northern part of Bangladesh, near the Meghalayan border of India. There are five main rivers, such as Kangsha, Someshawri, Dhala, Magra, and Teorkhali, passing through Netrokona. This district is also a part of the Surma-Meghna River System. All wetlands in Mohanganj are connected with the famous Kangsha River. Recently, wetland species at Mohanganj are remained in an extremely risky condition due to the diverse human activities as well as changes of LULC. The study of LULC, an important determinant of biodiversity in Mohanganj, is yet to be known. Therefore, this study was undertaken to unveil the LULC of Mohanganj Upazila over the last two decades, to know the wetland ecosystems of this Upazila over the last 5 years to determine the biodiversity changes and to assess the environmental threats using public perception.

## 2. Materials and Methods

### 2.1. Study Areas

**Figure 1:**
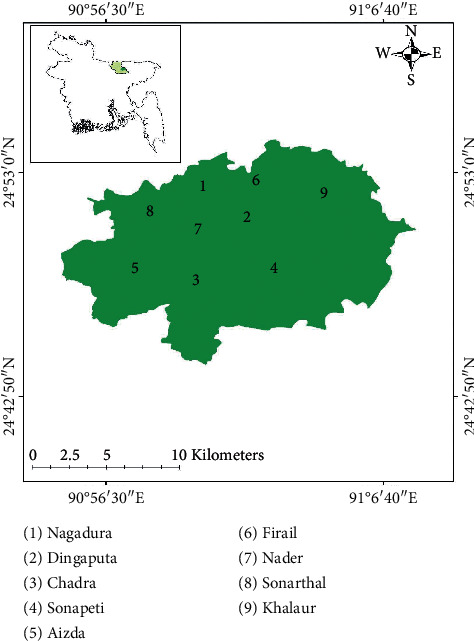
Sites of study area on different wetland biodiversity.

The survey was conducted at nine wetland ecosystems (Nagadura, Dingaputa, Chadra, Sonapeti, Aizda, Firail, Nader, Sonarthal, and Khalaura) at Mohanganj Upazilla (24°52′21″N 90°58′32″E) under the District of Netrokona, Bangladesh. The sites of the study are illustrated in Figure 1.

### 2.2. Land Use and Land Cover (LULC) Changes

**Figure 2:**
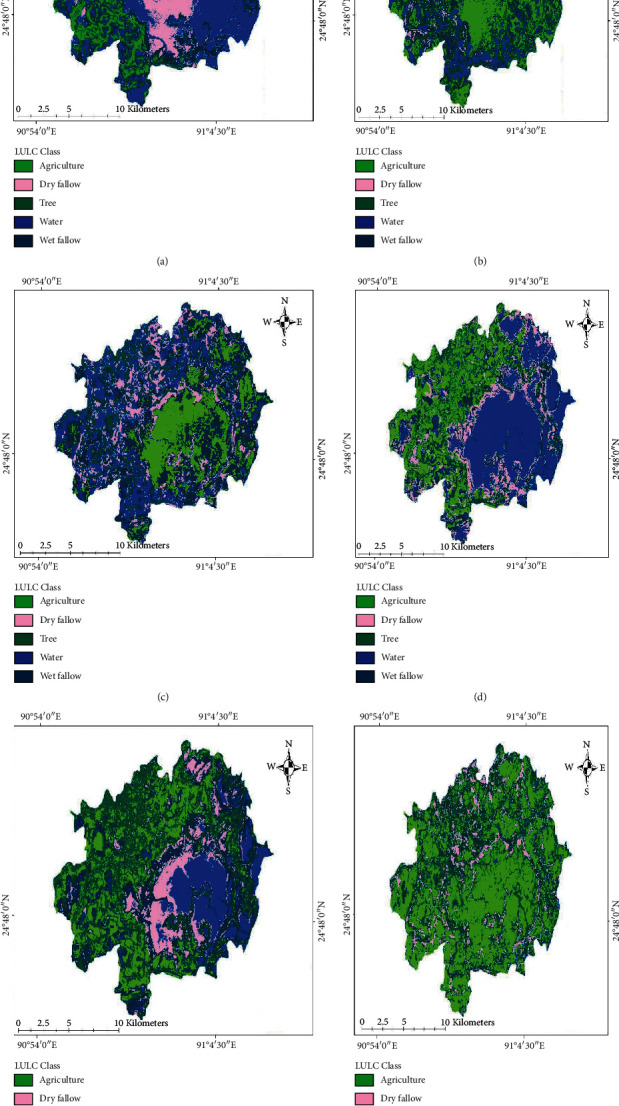
Changes of land use and land cover (LULC) from 2000 to 2020 in wetland based ecosystem of Mohanganj Upazila. (a) November, 2000, (b) February, 2000, (c) February, 2010, (d) November, 2010, (e) November, 2018, and (f) March, 2020.

**Figure 3:**
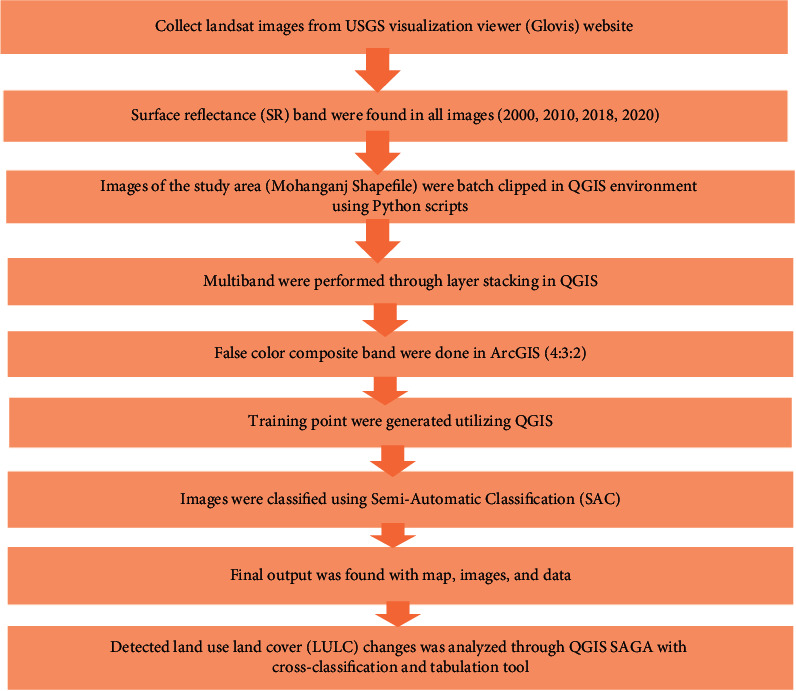
Flow chart highlights the procedure of land use and land cover (LULC) changes at wetland based ecosystems of Mohanganj Upazila.

Changes in agriculture, trees, water bodies, and other lands in Mohanganj Upazilla were analyzed through QGIS SAGA using cross-classification and tabulation tools during 2000, 2010, 2018, and 2020 (Figures 2(a)–2(f)). The procedure of LULC changes is highlighted in Figure 3.

### 2.3. Inception Meeting with Wetland Communities

An inception meeting was arranged at the office of the Department of Agricultural Extension (DAE) of Mohanganj Upazila. This meeting was conducted with community people, visitors, government officials, scientists, and fishermen who have been living in the surrounding areas of these wetland ecosystems for the last 20 years. The government officials were concerned regarding the effects of environmental threats on wetland biodiversity. In the meeting, the participants were briefed with the criteria of species to be considered as least concern (LC), vulnerable (VU), and endangered (EN) according to the guidelines of the International Union for the Conservation of Nature (IUCN). According to IUCN, a species was considered as vulnerable (VU) when it was likely to become endangered within the foreseeable future, a species was considered as endangered (EN) when it was at risk of extinction, and a species was considered as least concern (LC) when it has been categorized as a visible species.

### 2.4. Questionnaire

A survey questionnaire was developed considering the qualitative and quantitative data of least concern (LC), vulnerable (VU), and endangered (EN) species of fish, aquatic plants, and birds in the wetland ecosystem of Mohanganj. In addition, properties of all wetland ecosystems such as wetland area, water life, and topography, type of wetland, major crops, and causes of water body reduction are highlighted in Table 1.

### 2.5. Data Collection Procedure

We interviewed 50 people from each location. Among these, on average 10–12 people were women, and the rest of the people were men; both of them were 40–60 years old. The interviewees were involved in diverse professions, such as fishing, boating, industry, government officials, and research, whose average level of education was primary to graduate.

### 2.6. Record of Least Concerning, Vulnerable, and Endangered Species of Wetland Ecosystems

**Figure 4:**
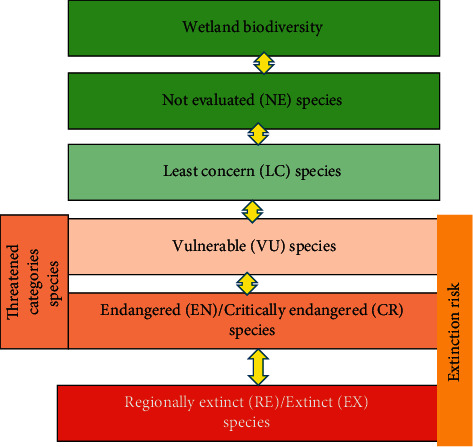
Flow chart of red list categories according to IUCN.

All the fish, aquatic, and birds species in the wetland ecosystems of Mohanganj were recorded during summer/raining (May to September) and winter/dry season (November to February) in 2015, 2017, and 2019. The species were recorded as LC, VU, and EN following the guidelines of [24] (Figure 4). Seasonal changes of fish, aquatic plant, and bird species categories (LC, VU, and EN) were recorded.

### 2.7. Binomial Probability

Binomial probability indicated the outcome of success or failure with multiple trials in an experiment. In this study, we used binomial probability to determine which species were more or less EN than other species. The probability of a species being EN was 0.1955 (41 EN species out of 210 total species). Binomial distribution was calculated using Minitab (Version 9.2).

### 2.8. Record of Major Environmental Threats

Major environmental threats and their impacts on fish, aquatic plants, and bird species in the wetland ecosystem services were noted based on the public perception to achieve sustainable development goals in Bangladesh.

### 2.9. Statistical Analysis

Seasonal variations of LC, VU, and EN fishes, aquatic plants, and bird species were analyzed in 2015, 2017, and 2019 using MS-Excel 2013. Land use and land cover (LULC) changes were analyzed through QGIS SAGA using a cross-classification and tabulation tool.

## 3. Results

### 3.1. Properties of Wetlands

The area under the wetland ecosystem was varied in winter and rainy seasons. In the rainy season of 2019, the wetland areas of Nagadura, Dingaputa, Chadra, Sonapeti, Aizda, Firail, Nader, Sonarthal, and Khalaura were 26, 46539, 90, 65, 59, 63, 105, 65, and 66 hectares, respectively, which was about 20% reduced during the winter/dry season. Rainfall was the main source of water in all wetlands. The topography of the surveyed wetland was flat to medium high. Rice was the main crop that was irrigated by using the water of these wetlands during the winter season. However, the irrigation during the rainy season was rain-fed (Table 1).

### 3.2. Land Use and Land Cover (LULC) Changes

Total area with agricultural crops was 2905.8, 7566.3, 3465.8, 5789.0, 4035.4, and 9653.5 ha in 2000 (Nov), 2010 (Nov), 2018 (Dec), 2000 (Feb), 2010 (Feb), and 2020 (March), respectively. The agricultural cropland area was increased from 2000 to 2010 in November. In contrast, cropland was decreased from 2000 to 2010 in February. Overall, increases in agricultural cropland were 19.3 to 66.7% from 2000 (November) to 2020 (March) (Figures 2(a)–2(f) and Table 2). Areas covered with trees were 5551.2, 4038.93, 8876.25, 8228.88, 3384.99, and 9810.63 ha in 2000 (November), 2010 (November), 2018 (December), 2000 (February), 2010 (February), and 2020 (March), respectively. Tree area was changed 27.2 to 119.7% from 2000 to 2020 (Figures 2(a)–2(f) and Table 2). The estimated water body was 7696.71, 5007.42, 3137.04, 2627.64, 4597.92, and 195.21 ha in 2000 (February), 2010 (February), 2020 (March), 2000 (November), 2010 (November), and 2018 (December), respectively. The water body was varied from 35 to 95.7% during the year 2000 to 2020 (Figures 2(a)–2(f) and Table 2). The dry area was covered with 1162.8 to 3643.2 ha from 2000 to 2020. The change in the dry area was 7 to 165% from 2000 to 2020 (Figures 2(a)–2(f) and Table 2). The total wet fellow was 2115.2 to 8991.9 ha recorded in 2000 to 2020. In this time, changes of wet fellow were 15.5 to 76.5% (Figures 2(a)–2(f) and Table 2).

### 3.3. Least Concern (LC), Vulnerable (VU), and Endangered (EN) Species

**Figure 5:**
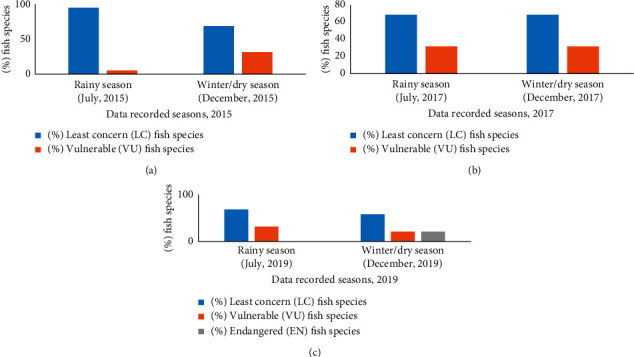
Yearly variation of fish species (a) 2015, (b) 2017, and (c) 2019 at different wetland ecosystems.

Among the 19 fish species studied from 2015 to 2019, 6 fish species (*Wallago attu, Ompok pabda, Channa punctate, Chitala chitala, Salmostoma phulo,* and *Corica soborna*) were found VU. However, chital (*Ch. chitala*) was found VU since rainy season, 2015. Among these fish species, 4 species (*W. attu, O. pabda, C. punctate,* and *Ch. chitala*) were EN category in winter, 2019. A total of 13 fish species were recorded as LC from 2015 to 2019 (Table 3). The percentage of LC fish species was reduced though from 2015 to 2019. In dry season 2019, about 21% of fish species were found EN with 21% VU and 58% LC category. Meanwhile, none of the fish species were recorded EN before the dry season, 2019 (Figures 5(a)–5(c)).

**Figure 6:**
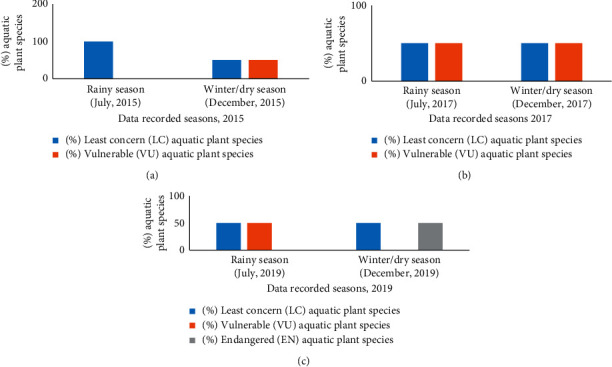
Yearly variation of aquatic plant species (a) 2015, (b) 2017, and (c) 2019 at different wetland ecosystems.

Among the 4 aquatic plant species, both common water hyacinth (*Eichhornia crassipes*) and water cabbage/water lettuce (*Pistia stratiotes*) were recorded as LC from the rainy season, 2015, to the winter season, 2019. However, 2 aquatic plant species, red and blue water lily (*Nymphaea nouchali*) and white lotus or sacred lotus (*Nymphaea lotus*), were categorized as LC in the rainy season, 2015, but they were changed to VU in the winter season, 2015 until the rainy season, 2019, which again moved to EN category in dry season 2019 (Table 4). The percentage of aquatic plant species under LC was 100% in the rainy season, 2015, but 50% of them were shifted to VU category during the rainy season, 2019. Meanwhile, during the winter season, 2019, all the VU plant species were changed to EN (Figures 6(a)–6(c)).

**Figure 7:**
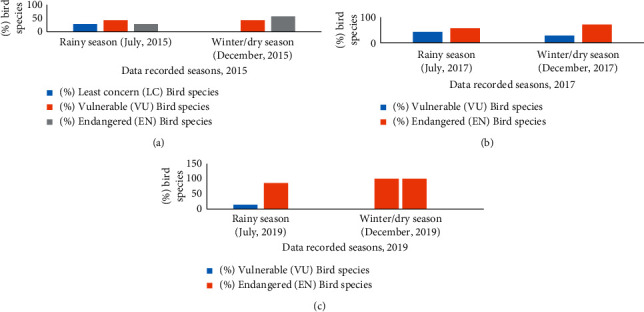
Yearly variation of bird species (a) 2015, (b) 2017, and (c) 2019 at different wetland ecosystems.

In the case of bird species, only two species such as Indian pond heron (*Ardeola grayii*) and common kingfisher (*Alcedo atthis*) were categorized as LC in the rainy season, 2015. However, within a short period *Ar. Grayii* was changed to EN category and *Alcedo atthis* was moved to VU category during the winter season, 2015, to the rainy season, 2019. Both wild duck (*Anas platyrhynchos*) and purple swamphen (*Porphyrio porphyria*) were grouped into VU during the rainy season, 2015, until winter season, 2019. Meanwhile, Black-headed gull (*Larus ridibundus*) and sukun (*Gyps bengalensis*) were categorized into EN from rainy season, 2015, till winter season, 2019. All the observed bird species (hundred percent) were found EN in the dry season, 2019 (Table 5; Figures 7(a)–7(c)).

### 3.4. Identification of Highly Endangered Species

We identified several species (fish, aquatic plants, and birds) more endangered (EN) than predicted by chance (Table 6). Among the bird species, purple swamphen (*Po. porphyria*) and black-headed gull (*L. ridibundus*) were highly endangered (*p* ≤ 0.001) than other bird species. Among the fish species, *W. attu* and *C. chitala* both were highly endangered (*p* ≤ 0.01) compared to the rest of the species. The aquatic plant species red and blue water lily (*Ny. nouchali*) was highly endangered (*p* ≤ 0.01) (Table 6).

### 3.5. Major Environmental Threats

**Figure 8:**
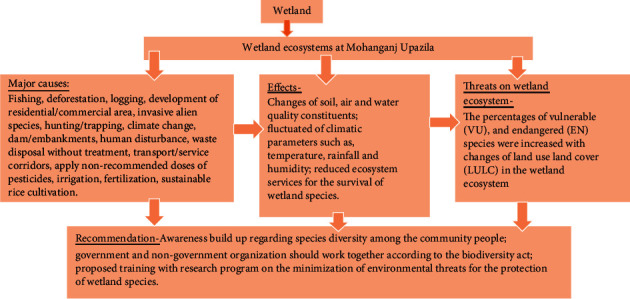
Effects of environmental threats on species diversity at wetland ecosystems of Mohanganj Upazila.

Based on public perception, major environmental threats in the study area were identified as unplanned fishing, deforestation, logging, random development of residential/commercial areas, invasive alien species, hunting/trapping, climate change, dam/embankments, human disturbance, waste disposal without treatment, transport/service corridors, application of nonrecommended doses of pesticides, unplanned irrigation during the dry season, chemical fertilization, and sustainable rice cultivation (Figure 8).

## 4. Discussion

Wetland is a good resource for biological diversity. Wetland supports aquatic birds, fishes, amphibians, reptiles, and plant species during important life stages by providing roosting, nesting, and feeding habitat as well as a sanctuary during extreme weather conditions [25].

In this study, the percentages of least concern (LC) fish species declined in 2019 as compared to 2015 and 2017. However, the percentage of both vulnerable (VU) and endangered (EN) fish species was increased in 2019. Interestingly, the vulnerability of fish species was increased in winter as compared to the summer season (Figures 5(a)–5(c)). Our study corroborates the finding of Hakaluki wetland in Bangladesh [26] where they found 14.46% and 21.69% fish species were recorded as VU and EN, respectively. According to the red list of IUCN, globally about 1% of fish species was critically endangered (CN), 6% of fish species were EN, 22% of fish species were VU, 30% of fish species were at Lower Risk Near Threatened (LRNT), and 30% fish species as lower risk least concerned (LRLC) [27]. Regionally, in the wetland of East Kolkata, about 59% of fish species were recorded as near threatened (NT) to EN [27].

Geographically, Mohanganj is occupied with a large river network and few big lakes, but excessive irrigation, low rainfall, and global warming reduce the water bodies of the wetland ecosystem (Table 1). As a result, only 57% of fish species were found to be LC, but 21.05% and 21.05% of fish species were recorded as VU and EN, respectively, during winter in 2019 (Figures 5(a)–5(c)). The fish species *Wallago attu, Ompok pabda, Channa punctate,* and *Chitala chitala* were recorded as an EN, but the family Carcharhinidae (e.g., *Wallago attu*) was found to be extremely EN (Table 6). Similar to our results, Chowdhury et al. [28] categorized 24, 19, 24, 7, and 3 fish species as available, moderately available, rarely available, very rarely available, and extinct, respectively among the 77 fish species under 25 families. Another study in Bangladesh reported that the highest number of fish species was observed from October to December; however, the lowest number of fish species was observed from March to April [29]. According to Islam et al. [29], out of 54 threatened red-listed fish species by IUCN in Bangladesh, only 30 species were highly visible for the last 20 years, but, currently, 23 species were categorized as only visible. Subsequently, a large number of fish species were added to the red list by the IUCN Red List of Bangladesh [30].

Aquatic plant species provide food and shelter for the survival of wetland fish species. In this study, both VU and EN aquatic plant species have been increased in 2019 as compared to 2015 and 2017, while 100% of aquatic plant species were found as LC during the rainy season in 2015. Meanwhile, over time LC species decreased and 50% of aquatic species were moved down to the EN category during winter in 2019. Also, the family Nymphaeaceae (species: *Nymphaea nouchali*) was recorded as highly EN species (Tables 4 and 6; Figures 6(a)–6(c)). In this study, it is proved that the diversity of wetland plant species has been declined in 2019 as compared to 2015. The decline of water bodies may be the principal reason for the decreasing trend of aquatic plant species in Mohanganj. Similar to these study areas, global water pollution is one of the major reasons for biodiversity losses in wetland ecosystems [31]. According to the public perception, the water bodies in the study area at Mohanganj Upazila were polluted due to excessive use of pesticides (Figure 8). Therefore, the loss of biodiversity in the wetlands of the study area in 2019 may happen due to water pollution by pesticides. In Asia, the number of wetland species, such as great knot (*Calidris tenuirostris*) and far eastern curlew (*Numenius madagascariensis*), was reduced rapidly due to water pollution [32].

All the bird species were recorded EN during winter in 2019 (Figures 7(a)–7(c) and Table 5) and the highly EN families were Rallidae (*Porphyrio porphyria*) and Laridae (*Larus ridibundus*) (Table 6). According to the red list of IUCN in Bangladesh [33], 38 bird species were near threatened (NT) and 78 species were considered threatened or near threatened. Among these bird species, dwarf kingfisher (*Ceyx erithaca*), bristled grassbird (*Chaetornis striata*), eagle (*Haliaeetus leucoryphus*), Indian spotted eagle (*Aquila hastate*), and masked finfoot (*Heliopais personata*) were globally identified as threatened. In Bangladesh, white-rumped vulture (*G. bengalensis*) and slender-billed vulture (*Gyps tenuirostris*) were extremely critical [33]. Also, 17 and 12 bird species were identified as VU and EN category, respectively, in Bangladesh [33]. Lesser adjutant (*Leptoptilos javanicus*), great hornbill (*Buceros bicornis*), and grey peacock-pheasant (*Polyplectron bicalcaratum*) were identified highly VU in Bangladesh [33]. In our study, we found that *G. bengalensis* was EN species in the wetland ecosystem of Mohanganj (Tables 5 and 6).

However, the land use and land cover (LULC) with crops, trees, waterbody, and dry and wet fallows is significantly important for the diversity of species in the wetland ecosystem. The change of biological resources in an ecosystem depends on the LULC of a particular territory. The LULC of Mohanganj Upazilla was changed in the last two decades due to human activities. Likewise, the LULC was changed across the globe. For instance, the water body was declined by 1% and the forest by 4% between 1990 and 1998 at Kibasira Swamp in Kilombero Valley of Tanzania [34]. There was a decrease in areas covered by water by 35% and forest by 9%, whereas *C. papyrus* L increased by 40% and cultivated land increased by 8% between 1998 and 2011 [34]. The LULC was sharply reduced in Inle Lake, Myanmar, which was 4.2 times higher in 2014 than that of 1989. LULC with forest area has been declined by 92 km^2^ (8.56%) for the last 25 years in the Inle wetland ecosystem [35]. Biodiversity with land use and land cover has been changed at Quirimbas National Park in Northern Mozambique of Africa due to intensive agriculture, human settlements, population growth, illegal exploitation of forest resources, and mining. Literature showed that about 86.95% of land use and land cover has been changed for 38 years. Total land area was decreased by about 301,761.7 ha, corresponding to 41.67% of the total coverage land [36]. It is estimated that almost 500 million hectares of wetlands are practically degraded in Africa. In those areas, 14% of the land degradation is a result of vegetation removal, 13% overexploitation, 49.5% overgrazing, and 24% agricultural practices [37]. Similar to world statistics, the LULC at Mohanganj Upazila with crops, trees, water bodies, and other natural resources has been reduced significantly in the last 20 years (Figures 2(a)–2(f) and Table 2). The main causes involved with LULC changes are anthropogenic factors, such as fires, territory fragmentation, intensification of agriculture, livestock, buildings, infrastructure, deforestation, urbanization, mining industry, and natural disasters [38].

In our study, the main reasons for LULC changes were unplanned fishing, deforestation, logging, random development of residential/commercial areas, invasive alien species, hunting/trapping, climate change, dam/embankments, human disturbance, waste disposal without treatment, transport/service corridors, application of non-recommended doses of pesticides, irrigation, fertilization, and sustainable agricultural production (Figure 8). According to the Millennium Ecosystem Assessment [MEA] [21] report, the loss of wetland habitats and land degradation were the consequences of overexploitation, alien invasive species, climate change, and pollution. The consequence of wetland declines resulted in a decline of LC species and the increase of VU and EN fish, bird, and aquatic plant species. In this context, *W. attu, N. nouchaii, G. bengalensis, P. porphyria,* and *L. ridibundus* were extremely endangered categories due to changes of land use and land cover (Table 5).

According to public perception, the loss of biodiversity in the wetlands is due to the rapid expansion of the sustainable agricultural system. Likewise, the expanded practice of sustainable agricultural systems is responsible for the reduction of biodiversity in the wetlands throughout the globe [39]. Intensification of agriculture results introduces new pests and pathogens in different cropping patterns [40] which needs an indiscriminate use of pesticides to control their resurgence. Thus, the wetland ecosystem is disrupted [41]. In the UK, agricultural intensification caused a massive reduction of biodiversity; two-thirds of 333 plant and animal species have been lost [42]. Sixty percent of the 1,146 freshwater taxa were assessed as threatened, and 228 species were reported to be extinct in wetlands since the last century in the world because of pollution [43, 44].

Wetlands are not only required for the conservation of biodiversity but also essential for food, fuel, water, climate regulation, aesthetic, spiritual, recreational activities, soil nutrient cycling, carbon sequestration, and sustainable livelihood for the human being [45]. Still, several aquatic fish and plant species such as *M. pancalus, P. puntio, H. fossilis, C. batrachus, L. rohita, M. vittatus, G. giuris, L. guntea, F. indicus, G. catla, C. nama, A. mola, E. crassipes,* and *P. stratiotes* are highly visible due to their high adaptation capacity with changing environment in this wetland ecosystem (Tables 3 and 4). As a result, community people are getting benefits from the wetland ecosystem for their sustainable livelihood. Therefore, the protection of wetland biodiversity is necessary for the mitigation of climate change and a sustainable environment. Hence, continuous research and monitoring, awareness building among the wetland community people, and collaborative research and development work between the government and nongovernment organizations are highly recommended for the protection of wetland ecosystems.

## 5. Conclusions

Wetland birds, aquatic plants, and fish species of Mohangonj Upazilla were recorded as VU to EN category between 2015 and 2019. The wetland species belonging to the EN category were considered as an extreme risk due to changes in land use and land cover. The decline of biodiversity was worsened in the dry season. *W. attu, N. nouchaii, G. bengalensis, P. porphyria,* and *L. ridibundus* were found in extremely endangered categories. If this condition prevails, then many of the wetland species will be extinct from Mohangonj in the next few years. The existing species should be restored for species conservation and environmental sustainability. Hence, awareness with training and research programs on the minimization of environmental threats will be helpful for the protection of wetland species to the development of a sustainable environment among the wetland-based community people.

## Figures and Tables

**Table 1:** Properties of different wetland ecosystems at Mohanganj Upazila.

Sl. number	Name of wetland	Average area of water body during the rainy season (hectare)	Average area of water body during the winter season (hectare)	% Water body reduced in winter season	Source of water	Water life	Topography of surrounding area	Major crops	Type of wetland	Causes for the reduction of water body during the winter season
1	Nagadura	26	6	23	Rainfall	Medium	Flat	Rice	Natural	Embankment, irrigation, low rainfall, and water transfer into another area for sustainable rice farming
2	Dingaputa	46539	6272	13	Rainfall	High	Flat	Rice	Natural	
3	Chadra	90	26	29	Rainfall	Medium	Medium high	Rice	Natural	
4	Sonapeti	65	22	34	Rainfall	Medium	Medium high	Rice	Natural	
5	Aizda	59	6	10	Rainfall	Low	Medium high	Rice	Natural	
6	Firail	63	18	29	Rainfall	Medium	Medium high	Rice	Natural	
7	Nader	105	19	18	Rainfall	Medium	Medium high	Rice	Natural	
8	Sonarthal	65	22	34	Rainfall	Medium	Medium high	Rice	Natural	
9	Khalaura	66	18	27	Rainfall	Low	Medium high	Rice	Natural	

**Table 2:** Changes of land use and land cover (LULC) from 2000 to 2020 in wetland ecosystems of Mohanganj Upazila.

LULC	Area (ha)			% Change			Area (ha)			% Change		
	2000 Nov	2010 Nov	2018 Dec	2000 Nov–2010 Nov	2010 Nov–2018 Dec	2000 Nov–2018 Dec	2000 Feb	2010 Feb	2020 March	2000 Feb–2010 Feb	2010Feb–2020 M	2000 Feb–2020 M
Agriculture	2905.8	7566.3	3465.8	160.3	−54.2	19.3	5789.0	4035.4	9653.5	−30	139.2	66.7
Tree	5551.2	4038.9	8876.2	−27.2	119.7	59.9	8228.8	3384.9	9810.6	−58.8	189.8	19.2
Water	7696.7	5007.4	3137.0	−35	−37	−60	2627.6	4597.9	195.2	74.9	−95.7	−92.5
Dry fallow	3389.7	3643.2	2634.8	7	−27.7	−22.6	1162.8	3081.9	2309.7	165	−25	98.6
Wet fallow	4540.9	3836.1	5970.5	−15.5	55.6	31.5	6276.0	8991.9	2115.2	43.2	−76.5	−66.2

**Table 3:** Seasonal variation of fish species at different wetland ecosystems.

Name of species	Local name	English name	Scientific name	Status of species, 2015		Status of species, 2017		Status of species, 2019	
				Rainy season (July 2015)	Winter/dry season (December 2015)	Rainy season (July 2017)	Winter/dry season (December 2017)	Rainy season (July 2019)	Winter/dry season (December 2019)
Fish	Chikra	Barred spiny eel	*Macrognathus pancalus*	LC	*LC*	LC	*LC*	LC	*LC*
	Puti	Puntio barb	*Puntius puntio*	LC	*LC*	LC	*LC*	LC	*LC*
	Shing	Stinging catfish	*Heteropneustes fossilis*	LC	*LC*	LC	*LC*	LC	*LC*
	Magur	Walking catfish	*Clarias batrachus*	LC	*LC*	LC	*LC*	LC	*LC*
	Boal	Freshwater shark	*Wallago attu*	LC	*VU*	VU	*VU*	VU	*EN*
	Rui	Rohu	*Labeo rohita*	LC	*LC*	LC	*LC*	LC	*LC*
	Koi	Climbing perch	*Anabas testudineus*	LC	*LC*	LC	*LC*	LC	*VU*
	Pabda	Pabdah catfish	*Ompok pabda*	LC	*VU*	VU	*VU*	VU	*EN*
	Tengra	Striped dwarf catfish	*Mystus vittatus*	LC	*LC*	LC	*LC*	LC	*LC*
	Bailla	Tank goby	*Glossogobius giuris*	LC	*LC*	LC	*LC*	LC	*VU*
	Gutum	Guntea loach	*Lepidocephalichthys guntea*	LC	*LC*	LC	*LC*	LC	*LC*
	Chingri	Shrimp	*Fenneropenaeus indicus*	LC	*LC*	LC	*LC*	LC	*LC*
	Katal	Catla	*Gibelion catla*	LC	*LC*	LC	*LC*	LC	*LC*
	Taki	Spotted snakehead	*Channa punctate*	LC	*VU*	VU	*VU*	VU	*EN*
	Chital	Clown knifefish	*Chitala chitala*	VU	*VU*	VU	*VU*	VU	*EN*
	Chada	Elongate glass perchlet	*Chanda nama*	LC	*LC*	LC	*LC*	LC	*LC*
	Chela	Finescale razorbelly minnow	*Salmostoma phulo*	LC	*VU*	VU	*VU*	VU	*VU*
	Mola	Mola carplet	*Amblypharyngodon mola*	LC	*LC*	LC	*LC*	LC	*LC*
	Kachki	Ganges river sprat	*Corica soborna*	LC	*VU*	VU	*VU*	VU	*VU*

Note: VU, vulnerable (any species that is likely to become endangered within the foreseeable future); EN, endangered (any *species* that is at risk of extinction); *LC,* least concern (a species that has been categorized by the International Union for Conservation of Nature [IUCN] as evaluated as not being a focus of *species* conservation).

**Table 4:** Seasonal variation of aquatic plant species at different wetland ecosystems.

Name of species	Local name	English name	Scientific name	Status of species, 2015		Status of species, 2017		Status of species, 2019	
				Rainy season (July 2015)	Winter/dry season (December 2015)	Rainy season (July 2017)	Winter/dry season (December 2017)	Rainy season (July 2019)	Winter/dry season (December 2019)
Aquatic plant	Kachuripana	Common water hyacinth	*Eichhornia crassipes*	LC	*LC*	LC	*LC*	LC	*LC*
	Topapana	Water cabbage/water lettuce	*Pistia stratiotes*	LC	*LC*	LC	*LC*	LC	*LC*
	Shapla	Red and blue water lily	*Nymphaea nouchali*	LC	*VU*	VU	*VU*	VU	*EN*
	Padma	White lotus or sacred lotus	*Nymphaea lotus*	LC	*VU*	VU	*VU*	VU	*EN*

Note: VU, vulnerable, (any *species* that is likely to become *endangered* within the foreseeable future); EN, endangered (any *species* that is at risk of extinction); LC, least concern (a species that has been categorized by the International Union for Conservation of Nature [IUCN] as evaluated as not being a focus of *species* conservation).

**Table 5:** Seasonal variation of bird's species at different wetland ecosystems.

Name of species	Local name	English name	Scientific name	Status of species, 2015		Status of species, 2017		Status of species, 2019	
				Rainy season (July 2015)	Winter/dry season (December 2015)	Rainy season (July 2017)	Winter/dry season (December 2017)	Rainy season (July 2019)	Winter/dry season (December 2019)
Bird	Bali Hash	Wild duck	*Anas platyrhynchos*	VU	*VU*	VU	*EN*	EN	*EN*
	Bog	Indian pond heron	*Ardeola grayii*	LC	*EN*	EN	*EN*	EN	*EN*
	Shukun	Bengal vulture	*Gyps bengalensis*	EN	*EN*	EN	*EN*	EN	*EN*
	Machranga	Common kingfisher	*Alcedo atthis*	LC	*VU*	VU	*VU*	VU	*EN*
	Pankouri	Indian shag	*Phalacrocorax fuscicollis*	VU	*VU*	VU	*VU*	*EN*	*EN*
	Kalim Bird	Purple swamphen	*Porphyrio porphyria*	VU	*EN*	EN	*EN*	*EN*	*EN*
	Gangchil	Black-headed gull	*Larus ridibundus*	EN	*EN*	EN	*EN*	*EN*	*EN*

Note: VU, vulnerable (any *species* that is likely to become *endangered* within the foreseeable future); EN, endangered (any *species* that is at risk of extinction); LC, least concern (a species that has been categorized by the International Union for Conservation of Nature [IUCN] as evaluated as not being a focus of *species* conservation).

**Table 6:** Endangered fishes, aquatic plants, and bird's species under families at different wetland ecosystems in 2019.

Category of species	Family	English name	Scientific name	Endangered families, 2019			
				No. of species in the family	No. of species endangered	The proportion of species endangered	Binomial probability
Fishes	Carcharhinidae	Freshwater shark	*Wallago attu*	4	4	1.00	0.00144^*∗∗*^
	Siluridae	Pabdah catfish	*Ompok pabda*	3	2	0.67	0.0918
	Channidae	Spotted snakehead	*Channa punctate*	6	2	0.33	0.239
	Notopteridae	Clown knifefish	*Chitala chitala*	4	3	0.75	0.023^*∗*^
Aquatic plants	Nymphaeaceae	Red and blue water lily	*Nymphaea nouchali*	7	5	0.71	0.0038^*∗∗*^
Birds	Anatidae	Wild duck	*Anas platyrhynchos*	3	2	0.67	0.091
	Ardeidae	Indian pond heron	*Ardeola grayii*	4	1	0.25	0.4
	Accipitridae	Bengal vulture	*Gyps bengalensis*	6	5	0.83	0.0013^*∗∗*^
	Alcedinidae	Common kingfisher	*Alcedo atthis*	4	2	0.50	0.147
	Phalacrocoracidae	Indian shag	*Phalacrocorax fuscicollis*	3	2	0.67	0.091
	Rallidae	Purple swamphen	*Porphyrio porphyria*	9	7	0.78	0.00025^*∗∗∗*^
	Laridae	Black headed gull	*Larus ridibundus*	7	6	0.86	0.0003^*∗∗∗*^
		Total			41	8.02	

Note: Even probability *P* = total proportion of species endangered/total number of species endangered = 8.02/41 = 0.1955. Binomial probability of each endangered species was calculated using statistical software Minitab. ^∗∗∗^ indicates significant difference at *p* ≤ 0.001 level of significance, ^∗∗^ indicates significant difference at *p* ≤ 0.01 level of significance, and ^∗^ indicate significant difference at *p* *≤* *0.05* level of significance.

## Data Availability

All the data are available within the manuscript.
